# Donkey genome and insight into the imprinting of fast karyotype evolution

**DOI:** 10.1038/srep14106

**Published:** 2015-09-16

**Authors:** Jinlong Huang, Yiping Zhao, Dongyi Bai, Wunierfu Shiraigol, Bei Li, Lihua Yang, Jing Wu, Wuyundalai Bao, Xiujuan Ren, Burenqiqige Jin, Qinan Zhao, Anaer Li, Sarula Bao, Wuyingga Bao, Zhencun Xing, Aoruga An, Yahan Gao, Ruiyuan Wei, Yirugeletu Bao, Taoketao Bao, Haige Han, Haitang Bai, Yanqing Bao, Yuhong Zhang, Dorjsuren Daidiikhuu, Wenjing Zhao, Shuyun Liu, Jinmei Ding, Weixing Ye, Fangmei Ding, Zikui Sun, Yixiang Shi, Yan Zhang, He Meng, Manglai Dugarjaviin

**Affiliations:** 1 College of Animal Science, Inner Mongolia Agricultural University, 306 Zhaowuda Road, Hohhot 010018, P. R. China; 2 School of Agriculture and Biology, Shanghai Jiaotong University; Shanghai Key Laboratory of Veterinary Biotechnology, 800 Dongchuan Road, Shanghai 200240, P. R. China; 3 Shanghai Personal Biotechnology Limited Company, 218 Yindu Road, Shanghai 200231, P. R. China; 4 SRA Inc. 6003 Executive Blvd. Suite 400, Rockville, MD20852, USA

## Abstract

The donkey, like the horse, is a promising model for exploring karyotypic instability. We report the de novo whole-genome assemblies of the donkey and the Asiatic wild ass. Our results reflect the distinct characteristics of donkeys, including more effective energy metabolism and better immunity than horses. The donkey shows a steady demographic trajectory. We detected abundant satellite sequences in some inactive centromere regions but not in neocentromere regions, while ribosomal RNAs frequently emerged in neocentromere regions but not in the obsolete centromere regions. Expanded miRNA families and five newly discovered miRNA target genes involved in meiosis may be associated with fast karyotype evolution. APC/C, controlling sister chromatid segregation, cytokinesis, and the establishment of the G1 cell cycle phase were identified by analysis of miRNA targets and rapidly evolving genes.

Donkeys and horses are globally important livestock, representing the *Equus* genus^1^,^2^. Compared with horses, donkeys have superior physiological characteristics, such as a better immune capacity and more effective energy metabolism^3^,^4^. The relationship between these species is complicated and confusing. For example, these animals can mate and produce mules or hinnies despite being different species. Karyotypic diversification is more prominent in *Equus* species than in other mammals^5^,^6^, suggesting that the *Equus* genus is a promising model for exploring the dynamics of chromosomal evolution^7^. A puzzling phenomenon is the relatively high frequency of centromere repositioning events^8^ in *Equus*, as at least seven cases have occurred between donkeys and horses, with at least six further in the donkey^9^. In our previous study, we investigated the mechanism of chromosomal rearrangement, including Robertsonian translocations and local rearrangements, using de novo assembled genome sequences from Przewalski’s wild horse (*Equus przewalskii*) and the Mongolian horse (*Equus caballus*)^7^. These results suggest that analysis based on whole genome sequences is a delicate and powerful method for studying chromosomal evolution. Here, we report the whole-genome sequence and de novo genome assembly of the donkey and Asiatic wild ass. Using these quality genome sequences, we addressed two questions: (1) what are the demographic and phylogenomic histories accompanying the speciation and genomic adaptive evolution in these representative *Equus* species, and (2) what are the underlying genetic and epigenetic mechanisms of fast karyotype evolution and frequent centromere repositioning.

## Results

### Genome sequencing, assembly and annotation

The genome of one male donkey was sequenced and de novo assembled using a whole-genome shotgun strategy (Table 1). Eight paired-end libraries (a standard genomic library that was sequenced using paired-end reads with insert sizes of 400–1000 bp), one single-end library (insert size: 1.5–1.9 kbp), and eight mate-paired libraries (insert sizes: 3–15 kbp) were constructed for genome sequencing (Supplementary Table 1). Paired-end libraries were sequenced using the Illumina Miseq platform, the single-end library was sequenced using the Roche 454 FLX+ platform, and mate-paired libraries were sequenced using the Illumina Hiseq2000 platform. The total sequence coverage was approximately 42.4-fold (genome size: ∼2.36 Gb) (Supplementary Table 2). For the Asiatic wild ass, one paired-end library (insert size: 500 bp) was constructed and sequenced using the Illumina Hiseq2000 platform (12.1-fold; Supplementary Tables 3 and 4). High contiguity genome sequences from the donkey were generated after their de novo assembly, and they consisted of 2,166 scaffolds (>1k bp) with a total size of 2.36 Gb (Supplementary Table 5). The N50 lengths of the contigs and scaffolds were 66.7 kb and 3.8 Mb, respectively. Compared with other previously published genome sequences^10^,^11^,^12^,^13^ (Supplementary Figs 1 and 2), the contiguity of the contigs in the donkey assemblies was better. We also validated 248 core eukaryotic genes^14^ in the donkey genome assemblies and found considerable completeness (Supplementary Table 6). These improvements may be the result of longer lengths for the sequence reads, because the reads used in this study were mainly generated by the Illumina Miseq platform (2 × 251 bp) and longer than those generated by Hiseq2000 (2 × 100 bp)^10^,^11^,^12^,^13^.

To improve our gene prediction accuracy, eight types of tissue samples (heart, liver, spleen, lung, kidney, brain, spinal cord, and muscle) from another female donkey were used to construct a normalized cDNA library. RNA-seq was performed using the Roche 454 FLX+ platform, and 1,390,416 reads were generated with an average length of 522 bp (Supplementary Fig. 3 and Supplementary Table 7). Donkey genome annotation was performed using a dexterous genome annotation pipeline, including both ab initio predictions (Augustus and SNAP)^15^,^16^ and homology-based methods (RNA-seq of the female donkey, and homologous proteins sequences of the Thoroughbred horse^17^). A total of 23,214 protein-coding genes were predicted in the donkey genome (Table 1, Supplementary Figs 4 and 5) averaging 1,281 bp coding sequences (CDSs) per gene. Among these genes, 15,648 could be confirmed with the RNA-seq sequences (Supplementary Fig. 6).

### Demographic history and phylogenetic analysis

**Figure 1:**
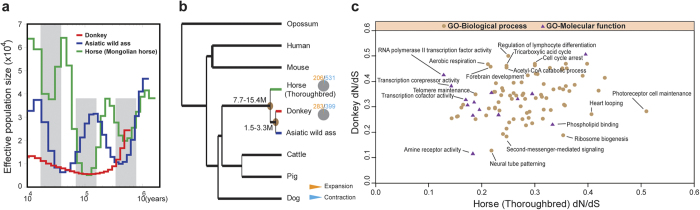
Analysis of evolution genomics. (**a**) Reconstructed population demographics of donkey, Asiatic wild ass and horse for the last 1 million years. (**b**) Phylogenetic tree of nine mammals. The numbers represent the time of divergence. The proportion of expanded and contracted gene families are shown as pie charts at branch termini. (**c**) Rapidly evolving functions of donkey and horse.

We identified 2,187,070 and 3,321,087 heterozygous SNPs (within each individual) in the donkey and the Asiatic wild ass genomes, respectively (Supplementary Table 8). The rate of heterozygosity was considerably higher in the Asiatic wild ass than in donkey. We also reconstructed the donkey, Asiatic wild ass, and horse population demographics over the last one million years (Fig. 1a). Because Thoroughbred horse (Twilight) pedigrees show substantial levels of inbreeding^17^, we used heterozygous SNPs from the Mongolian horse^7^. Our demographic analysis revealed three horse population bottlenecks, which is consistent with the quaternary glaciations. Similar to those of the horse, Asiatic wild ass lineages show extremely dynamic demographic trajectories. Interestingly, the size of the donkey population was steady. We believe that this stability is because the donkey ancestors (African wild asses) living in northeast Africa^2^ may have been influenced by different climates during the quaternary glaciations, as climate changes could result in grassland contraction or expansion^18^.

The rich *Equus* fossil records have made this genus a model for evolutionary processes^19^. Previous research has shown that the donkey and the horse shares common ancestors approximately 6.4–12.7 million years ago^20^,^21^,^22^. In this paper, we constructed a phylogenetic tree using 5,665 single-copy orthologs from nine species^17^,^23^,^24^,^25^,^26^,^27^,^28^ (Fig. 1b). As shown in this tree, the Asiatic wild ass is most closely related to the donkey, and together they form a sister group with the horse. Our results show that the donkey separated from the horse lineage approximately 7.7–15.4 million years ago, whereas the donkey and the Asiatic wild ass diverged approximately 1.5–3.3 million years ago. These estimates are comparable to the earliest divergence times reported^20^,^21^,^22^.

### Genetic evolution

To obtain greater insight into the evolutionary dynamics of these genes, we calculated the expansion and contraction of orthologous gene clusters between the donkey and the horse. A total of 283 gene families in the donkey showed significant expansion (P < 0.05) compared with 206 in the horse (Fig. 1b). The functional categories that were enriched in significant donkey gene family expansions included olfactory transduction (KEGG:map04740, p = 5.355e–08) and protein digestion and absorption (KEGG:map04974, p = 0.01327) (Supplementary Table 9). The horse gene family expansions were primarily associated with defense responses (GO:0006952, p = 0.011693853) and responses to stress (GO:0006950, p = 0.028023107) (Supplementary Table 10).

Rapidly evolving genes are one of the primary contributors to such functional changes. We identified 1,292 genes evolving significantly (p < 0.05) faster in the donkey than in the horse, and 706 genes evolving significantly (p < 0.05) faster in the horse than in the donkey. Rapidly evolving genes in domestic donkeys are significantly associated with aerobic respiration (GO:0009060, p = 0.027964968), forebrain development (GO:0030900, p = 0.006710136), regulation of lymphocyte differentiation (GO:0045619, p = 0.024669795), the tricarboxylic acid cycle (GO:0006099, p = 0.016761429), and the acetyl-CoA catabolic process (GO:0046356, p = 0.016761429) (Fig. 1c, Supplementary Table 11). These changes may be correlated with more effective energy metabolism^4^ and improved immune capacity in donkeys compared with horses. More specifically, twenty genes that are associated with forebrain development were found to be rapidly evolving in the donkey. Also, cell cycle arrest (GO:0007050, p = 0.020903) and telomere maintenance (GO:0000723, p = 0.002098) are rapidly evolving in the donkey genome, which may be associated with rapid karyotypic evolution. In contrast, rapidly evolving genes in the horse are significantly enriched in second-messenger-mediated signaling (GO:0019932, p = 0.001296105), heart looping (GO:0001947, p = 0.046821101), neural tube patterning (GO:0021532, p = 0.041550593), photoreceptor cell maintenance (GO:0045494, p = 0.041550593), and ribosome biogenesis (GO:0042254, p = 0.035248368) (Fig. 1c, Supplementary Table 12). These results may be associated with the animated disposition and greater athletic ability of the horse.

### Synteny analysis and repetitive sequences

**Figure 2:**
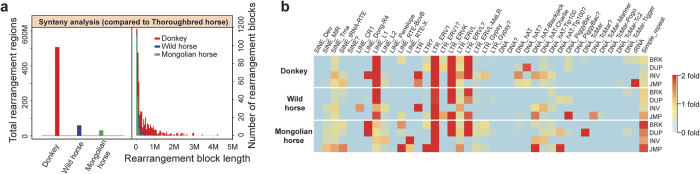
Whole genome synteny analysis. Comparisons of the donkey, wild horse and Mongolian horse genomes to the Thoroughbred horse genome. (**a**) The number of rearrangement blocks in donkey, wild horse, Mongolian horse genomes with respect to the Thoroughbred genome. (**b**) The content of some repetitive sequences significantly increased in rearrangement regions compared with the collinearity region.

Dramatic chromosomal rearrangement in *Equus* individuals is a notable characteristic compared with other mammals^5^,^6^. However, genome-wide rearrangements between the donkey and horse have not been characterized given that donkeys have a different number of chromosomes (2n = 62) than horses (2n = 64)^5^. We performed whole-genome synteny analysis between the donkey and Thoroughbred horse genomes. A collinearity region between the donkey and Thoroughbred horse was approximately 1.89 Gb (Fig. 2a). Four types of rearrangements, BRK (insertion of unknown origin), DUP (inserted duplication), INV (inversion), and JMP (relocation) were identified. Rearrangement of the donkey genome was particularly evident when donkey-Thoroughbred horse genome alignments were compared with those of Thoroughbred horse-wild horse and Thoroughbred horse-Mongolian horse, as more large-scale chromosomal rearrangements can be found in the donkey genome.

Previous research has indicated that repetitive sequences are associated with syntenic breakpoints and chromosomal fragility. Seven types of common repetitive sequences in the donkey genome were identified: short interspersed repeated sequences (SINEs), long interspersed repeated sequences (LINEs), long terminal repeats (LTRs), DNA elements, satellites, simple repeats, and low complexity. Overall, analyses of these sequences indicated that 42% of the donkey genome sequences are repetitive sequences (Supplementary Table 13), which is comparable to the horse (41.4%). Satellite sequences comprise 0.05% of the donkey genome, which is considerably lower than in the horse (1.59%). Satellite sequences are often associated with centromeres^26^, including some that are new in the donkey. The proportions of LINE_L1 and LTR_ERV1 increased, but those of LINE_L2 and several other repetitive sequences decreased in breakpoint regions (Fig. 2b). This phenomenon is more evident in the donkey genome, which is consistent with our previous findings^7^.

### Chromosome rearrangements and sequence signatures in centromere regions

**Figure 3:**
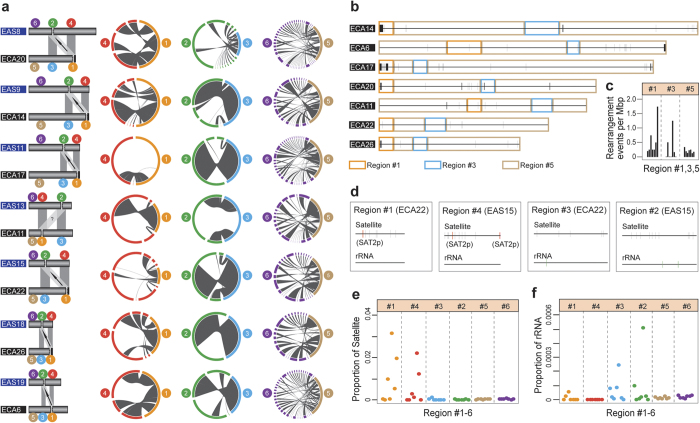
Chromosomal rearrangements and characteristic sequences in centromere regions. (**a**) Landscape of chromosomal rearrangements. Column 1: Six regions categorized in donkey and horse chromosomes. They are: #1(orange): Centromere regions in the horse chromosome; #2(green): Centromere regions in the donkey chromosome (at least 6 centromeres are neocentromeres); #3(blue): Homologous regions in the horse chromosome related to region #2; #4(red): Homologous regions in the donkey chromosome related to region #1; #5(brown): Other regions in the horse chromosome; #6(purple): Other regions in the donkey chromosome. (EAS: Equus asinus; ECA:Equus caballus). The arrow indicates the direction of two corresponding centromere repositionings. The question mark (‘?’) indicates the direction of two corresponding centromere repositionings that are not classified. Column 2: Synteny analysis between region #4 and region #1. Column 3: Synteny analysis between region #2 and region #3. Column 4: Synteny analysis between region #6 and region #5. (**b**) Chromosomal rearrangements between donkey and Thoroughbred horse. Black vertical lines represent rearrangement regions in the Thoroughbred horse chromosomes. (**c**) Numbers of rearrangements events in seven pairs of chromosomes. (**d**) Distribution of satellite sequences and ribosomal RNA in region #1, #3 of ECA22 and region #2, #4 of EAS15. (**e**) Proportion of satellite sequences in regions #1–6. (**f**) Proportion of ribosomal RNA in regions #1–6.

A striking phenomenon in *Equus* is the relatively high frequency of centromere repositioning events^9^. Although such events provide a potentially powerful evolutionary force for reproductive isolation and speciation, the underlying mechanisms remain unclear^29^. Comparative FISH studies have found that at least seven different centromere repositioning events occurred between the donkey and horse, and at least six further occurred in the donkey alone^9^. Based on the quality of whole-genome donkey sequences, we were able to perform microscopic analyses across the normal centromere regions of the horse (Thoroughbred) as well as neocentromere regions and inactive centromere regions in donkeys. Using the same probes^9^ and two major *Equus* satellite sequences^30^ as in previous studies, we identified the centromere regions of donkey and horse chromosomes. Six types of regions were categorized into seven pairs of chromosomes in donkeys and horses (Fig. 3a, Supplementary Table 14) including the following: (1) centromere regions in horse chromosomes (“region #1” hereafter), (2) centromere regions in donkey chromosomes (at least six centromeres are neocentromeres, region #2), (3) homologous regions in horse chromosomes related to region #2 (region #3), (4) homologous regions in donkey chromosomes related to region #1 (region #4), (5) other regions in horse chromosomes (region #5), (6) other regions in donkey chromosomes (region #6).

Any shift in centromeric function without chromosomal rearrangement can be considered centromere repositioning^30^. However, some researchers have noted that centromere regions are hot spots for chromosomal changes in evolution and disease^31^. We explored chromosomal rearrangements using synteny analysis based on genomic sequences between homologous regions (Fig. 3a) (region #1 vs. region #4, region #3 vs. region #2, region #5 vs. region #6). Increased rearrangements were detected in several chromosomes (Fig. 3b,c) when region #1 and region #4 (representing inactive centromere regions) were compared, and these were potentially caused by an accumulation or loss of satellite sequences. In contrast, no obvious increased rearrangements were detected (Fig. 3b,c) when region #3 and region #2 (representing neocentromere regions) were compared. Thus, we confirmed previous study results indicating that a DNA fragment can acquire centromere function without sequence alteration^30^,^32^.

To date, no prominent sequence characteristics have been confirmed to promote centromere repositioning, although it is widely accepted that neocentromeres can gradually accumulate satellite sequences accompanied by centromerization^29^. We noticed that the content of the satellite sequences in region #1 and region #4 in several chromosomes was increased (Fig. 3d,e, Supplementary Fig. 7). Twenty types of satellite sequences were examined in this study (Supplementary Fig. 8). Because region #1 of ECA14, ECA20, ECA22, ECA26 and region #4 of EAS8, EAS15 contained abundant SAT2p^30^ (Supplementary Fig. 8), we believe that the SAT2p were accumulated in the process of centromerization. Region #1 of ECA6, ECA11, and ECA17 does not contain abundant SAT2p, indicating these three centromeres may be novel. Unexpectedly, ribosomal RNAs were discovered in neocentromere regions and their homologous regions. In contrast, no ribosomal RNA could be detected in region #4 (inactive centromere regions) (Fig. 3d,f). A neighbor-joining tree constructed using conservative 5SrRNAs revealed that these 5SrRNAs are closely related (Supplementary Fig. 9). It is particularly worth mentioning that the ribosomal RNAs are located in the fibrillar centers of the nucleolus and play an important role in the organization of the nucleolus^33^. More research is needed to explain the genetic association between ribosomal RNA, nucleoli, and centromere repositioning.

### Small RNA-seq, and prediction of novel miRNA targets

**Figure 4:**
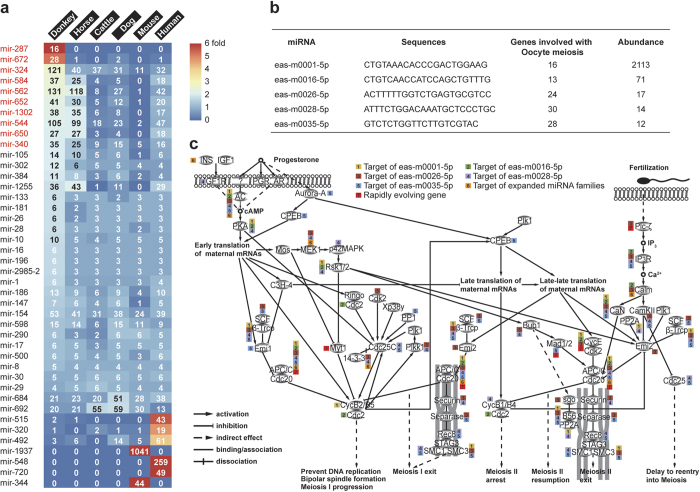
Novel miRNAs, expanded miRNA families and rapidly evolving genes in donkey, which are associated with the meiosis pathway. (**a**) Expanded miRNA families (red) in the donkey genome. (**b**) Five novel miRNAs targeting meiosis in the donkey genome identified by RNA-seq. (**c**) The donkey meiosis pathway. Small boxes indicate that the gene is regulated by novel miRNAs, expanded miRNA families or rapidly evolving genes.

To understand the role of epigenetic regulation in karyotypic evolution^29^, we annotated the non-coding RNAs and analyzed differentially expanded miRNA families in the donkey vs. other mammalian species. A total of 1198 miRNAs, 512 snoRNAs, 530 snRNAs, and 189 lncRNAs were identified in the donkey genome (Supplementary Table 15). The number of miRNAs in the donkey genome was comparable to humans (1215), mice (1497), and was higher than horses (881), dogs (647), and cattle (494)^34^. However, the distribution of miRNAs in the different miRNA families was quite different between donkeys and other mammals (Fig. 4a). We found that several miRNA families were expanded in the donkey genome (Fig. 4a, c). Targeted genes in donkey expanded miRNA families that were significantly enriched were related to the cell cycle, cancer, and oocyte meiosis, which are probably associated with fast karyotype evolution (Supplementary Table 16). From the small RNA libraries that were constructed from the above-mentioned eight types of tissue samples (Supplementary Table 17), we identified 118 miRNA families matching those in the existing miRNA database and 40 novel miRNAs specific to the donkey (Supplementary Table 18). Five of the newly discovered donkey miRNAs target genes are involved in meiosis (Fig. 4b,c, Supplementary Table 19), suggesting fast karyotype evolution. In the meiosis pathway, another five genes are rapidly evolving (Fig. 4c). APC/C, which controls sister chromatid segregation, cytokinesis, and establishment of the G1 phase of the cell cycle, was identified by analysis of miRNA target and rapidly evolving genes^35^,^36^.

## Discussion

The donkey and Asiatic wild ass genomes supplement the reference genome for the *Equus* genus. Our comparative analysis based on these genomic sequences provides important insight into the demographic history and adaptive evolution of *Equus*. In addition, these results enhance our understanding of the chromosomal rearrangements and dynamics of characteristic sequences associated with centromere repositioning. These data will be beneficial to future research of the genomics of the *Equus* genus and mammalian chromosomal evolution.

## Methods

### Sampling and genome sequencing

All animal care and research procedures were carried out in accordance with the guiding principles for the care and use of laboratory animals and were approved by the Institutional Animal Care and Use Committee at Inner Mongolia Agricultural University. For donkey genome sequencing, a 7-year-old male donkey was selected from the Xilingol League of Inner Mongolia, China on 18 February 2010. For Asiatic wild ass genome sequencing, approximately 5 ml of blood from a male Asiatic wild ass was provided by the Bayan Nur Forestry Administration. The blood sample was collected during veterinary exams for several Asiatic wild asses on 1 March 2002. No Asiatic wild ass was hurt or captured as a result of these studies. DNA was extracted from peripheral blood cells. Eight paired-end libraries (insert sizes: 400, 450, 700, and 1000 bp), one single-end library (insert size 1.5–1.9 kb), and eight mate-paired libraries (insert sizes: 3, 5, 8, 12, and 15 kb) were constructed for donkey genome sequencing. Paired-end libraries were sequenced using the Illumina Miseq platform (2 × 251 bp), the single-end library was sequenced using the Roche 454 FLX+ platform (average: 510 bp), and Mate-paired libraries were sequenced using the Illumina Hiseq2000 platform (2 × 100 bp). For the Asiatic wild ass, one paired-end library (insert size 500 bp) was constructed and sequenced using the Illumina Hiseq2000 platform (2 × 100 bp). Library preparation and sequencing followed the manufacturer’s instructions.

### Data filtering

Cutadapt1.2.1 (https://pypi.python.org/pypi/cutadapt/1.2.1) was used to trim adapter sequences from sequence reads generated by Illumina Miseq and Hiseq2000. Low-quality reads and reads with potential sequencing errors were also eliminated. For reads generated by Illumina Miseq, if the average phred quality scores for five consecutive bases were <Q20, we trimmed reads from the 3′-end. For reads generated by Illumina Hiseq2000, if the average phred quality scores of five consecutive bases were <Q20, we removed this read and its matching sequence.

### Donkey genome assembly

We first assembled the sequence reads of the pair-end and single-end libraries into contigs and scaffolds using Newbler v2.8. Then, we used SSPACE software^37^ and information for the mate-pair libraries to construct longer scaffolds. Finally, Gapcloser (http://soap.genomics.org.cn/soapdenovo.html) was used to close gaps inside scaffolds.

### Repetitive sequence and noncoding sequences analysis

RepeatMasker (http://www.repeatmasker.org/) was used to identify interspersed repeats and low complexity DNA sequences from the donkey and Thoroughbred horse genomes. Twenty types of satellite sequences were then plotted in a “heat map” using R software. 5SrRNA sequences were used to build the neighbor-joining tree using MEGA6^38^. Genome noncoding sequence annotation was used in the Rfam database^34^. A small RNA library was constructed from eight types of tissue samples (heart, liver, spleen, lung, kidney, brain, spinal cord, and muscle) from another female donkey. The library was sequenced using the Miseq platform. For novel miRNA identification, mireap (http://mireap.sourceforge.net/) was used. For target gene annotation, Miranda^39^ was used.

### Genome annotation and RNA-seq

Donkey genome annotation was performed using the MAKER^40^ annotation pipeline, which included ab initio predictions and homology-based methods. Ab initio predictions were performed using Augustus^15^ and SNAP^16^. cDNA data were generated from multiple RNA sources. cDNA libraries were constructed from eight types of tissue samples (heart, liver, spleen, lung, kidney, brain, spinal cord, and muscle) from another female donkey. The libraries were sequenced using the Roche 454 FLX+ platform. Homology-based prediction was performed by blasting against homologous protein sequences of Thoroughbred horse^17^ and cDNA sequences from donkey.

### Heterozygosity rate and demographic history

Qualified sequence reads from pair-end libraries of the donkey and Asiatic wild ass were mapped to the scaffolds of the donkey. SNPs and InDels were called using the Genome Analysis Toolkit^41^ following its manual. We flagged a candidate SNP as a likely false-positive if it exhibited the following characteristics: (1) sequence coverage at that point is more than 200 or less than 4; (2) HaplotypeScore >13.0, MQ < 40, QD < 2; (3) ReadPosRankSum <−8.0, MQRankSum <−12.5. The demographic histories of the donkey, Asiatic wild ass, and Mongolian horse were inferred using “pairwise sequentially Markovian coalescence” (PSMC)^42^ based on SNP distribution. Parameters were set as follows: −N30 −t15 −r5 −p 4+25*2+4+6. The *Equus* generation time (g) = 5 years and the neutral mutation rate per generation (μ) = 2.5 × 10^−8^ were set. Because low sequence coverage (below 20-fold) deeply impacted PSMC inference^42^, we performed a correction for Asiatic wild ass assuming a uniform False Negative Rate (uNFR = 26%) reported in previous research^19^.

### Phylogeny analysis

Protein-coding genes from seven mammalian species (opossum, dog, pig, cattle, Thoroughbred horse, mouse, and human) downloaded from Ensembl (http://www.ensembl.org) were used in addition to donkey genes to define gene families by OrthoMCL^43^. Thereafter, 5,665 single-copy families, which were generated from this analysis, were used to reconstruct phylogenies and estimate the time points of divergence. Protein-coding gene sequences from the Asiatic wild ass were generated by mapping reads from the Asiatic wild ass to the scaffolds of the donkey with samtools^44^ and genBlastG^45^. The protein sequences of orthologous gene sets were aligned by MUSCLE^46^ with its default settings. Poor alignment sites were eliminated using Gblock^47^. The phylogeny tree (including nine species) was drawn by PhyML^48^ using the JTT model. Based on the reconstructed phylogeny tree, we estimated the evolutionary time scales by PAML^49^. Calibration times were queried from the TimeTree database (http://www.timetree.org).

### Gene family expansion and contraction

Gene families were defined by OrthoMCL^43^. Gene family expansion analysis was performed by CAFE^50^ based on a reconstructed phylogeny tree.

### Rapidly evolving genes and dN/dS analysis

This analysis utilized 6,771 1:1 orthologous genes from seven species (donkey, Thoroughbred horse, dog, pig, cattle, mouse, and human). The protein sequences from orthologous gene sets were aligned by MUSCLE^46^ using default settings. Gblock^47^ was used to eliminate poor alignment sites. Afterward, dN/dS ratios for each gene were estimated with the codeml function in the PAML package^49^. The maximum-likelihood method was used to estimate dN (the rate of non-synonymous substitution), dS (the rate of synonymous substitution) and dN/dS (the ratio of non-synonymous substitutions to the rate of synonymous substitutions). The likelihood ratio test was used to evaluate the p-value for each gene.

### Synteny analysis and SV calling

We used Mauve Contig Mover^51^ to order donkey genome drafts relative to the Thoroughbred horse genome. Then, we used MUMmer^52^ to perform whole-genome synteny analysis. Genome rearrangements were identified using the nucmer module. The parameter was Options “-c 800 -g 300–l,100”.

## Additional Information

**Accession codes:** The Whole Genome Shotgun project has been deposited in DDBJ/EMBL/GenBank as project accession PRJNA200657 and PRJNA200654 of donkey and Asiatic wild ass, respectively. The genome assembly of donkey has been deposited at DDBJ/EMBL/GenBank under the accession JREZ00000000 and this version described in this paper is version JREZ01000000. Transcript sequencing data have been deposited under Short Read Archive (SRA) accession SRR1562259. Asiatic wild ass genome sequencing data have been deposited under Short Read Archive (SRA) accession SRR1562345.

**How to cite this article**: Huang, J. *et al.* Donkey genome and insight into the imprinting of fast karyotype evolution. *Sci. Rep.* **5**, 14106; doi: 10.1038/srep14106 (2015).

## Supplementary Material

Supplementary Information

## Figures and Tables

**Table 1:** Donkey genome assembly and structural annotation.

Total sequence length	2,357,920,133 bp
Total contig length	2,324,805,719 bp
Number of contigs >200 bp	71,732
N50 contig length	66,737 bp
Number of scaffolds >1 kb	2,166
N50 scaffolds length	3,803,025 bp
Average sequence depth	42.4×
GC content	41.28%
Protein-coding genes	23,214
