# Corrigendum: Donkey genome and insight into the imprinting of fast karyotype evolution

**DOI:** 10.1038/srep17124

**Published:** 2015-12-18

**Authors:** Jinlong Huang, Yiping Zhao, Dongyi Bai, Wunierfu Shiraigol, Bei Li, Lihua Yang, Jing Wu, Wuyundalai Bao, Xiujuan Ren, Burenqiqige Jin, Qinan Zhao, Anaer Li, Sarula Bao, Wuyingga Bao, Zhencun Xing, Aoruga An, Yahan Gao, Ruiyuan Wei, Yirugeletu Bao, Taoketao Bao, Haige Han, Haitang Bai, Yanqing Bao, Yuhong Zhang, Dorjsuren Daidiikhuu, Wenjing Zhao, Shuyun Liu, Jinmei Ding, Weixing Ye, Fangmei Ding, Zikui Sun, Yixiang Shi, Yan Zhang, He Meng, Manglai Dugarjaviin

Scientific Reports 5 Article number: 14106; 10.1038/srep14106 published online: 09 16 2015; updated: 12 18 2015.

In this Article, there are typographical errors in the accession numbers:

“The Whole Genome Shotgun project has been deposited in DDBJ/EMBL/GenBank as project accession PRJNA200657 and PRJNA200654 of donkey and Asiatic wild ass, respectively.”

should read:

“The Whole Genome Shotgun project has been deposited in DDBJ/EMBL/GenBank as project accession PRJNA259598 and PRJNA259601 of donkey and Asiatic wild ass, respectively.”

