# Supplementary material for: Donkey genome and insight into the imprinting of fast karyotype evolution
Source: Sci Rep. 2015 Sep 16;5:14106. doi: 10.1038/srep14106 (PMC4571621; doi:10.1038/srep14106)
Supplement: Supplementary Information [file srep14106-s1.pdf]

Supplementary information for:

Donkey genome and insight into the imprinting of fast karyotype evolution

Jinlong Huang[1]†, Yiping Zhao[1]†, Dongyi Bai[1]†, Wunierfu Shiraigol[1]†, Bei Li[1]†, Lihua Yang[1]†, Jing Wu[1]†, Wuyundalai Bao[1]†, Xiujuan Ren[1], Burenqiqige Jin[1], Qinan Zhao[1], Anaer Li[1], Sarula Bao[1], Wuyingga Bao[1], Zhencun Xing[1], Aoruga An[1], Yahan Gao[1], Ruiyuan Wei[1], Yirugeletu Bao[1], Taoketao Bao[1], Haige Han[1], Haitang Bai[1], Yanqing Bao[1], Yuhong Zhang[1], Dorjsuren Daidiikhuu[1], Wenjing Zhao[2], Shuyun Liu[2], Jinmei Ding[2], Weixing Ye[3], Fangmei Ding[3], Zikui Sun[3], Yixiang Shi[3], Yan Zhang[4]*, He Meng[2]*, Manglai Dugarjaviin[1]*

[1]College of Animal Science, Inner Mongolia Agricultural University, 306 Zhaowuda Road, Hohhot 010018, P.R. China.

[2]School of Agriculture and Biology, Shanghai Jiaotong University; Shanghai Key Laboratory of Veterinary Biotechnology, 800 Dongchuan Road, Shanghai 200240, P. R. China.

[3]Shanghai Personal Biotechnology Limited Company, 218 Yindu Road, Shanghai 200231, P.R. China.

[4]SRA Inc. 6003 Executive Blvd. Suite 400, Rockville, MD20852, USA.

† These authors contributed equally to this work.

* For correspondence: Manglai Dugarjaviin (dmanglai@imau.edu.cn); He Meng (menghe@sjtu.edu.cn); Yan Zhang (yan_zhang@sra.com).

Supplementary Figure 1. Comparison of the size of contigs and scaffolds for the donkey genome assembly with other genome assemblies. The arrow indicates the donkey genome assembly.

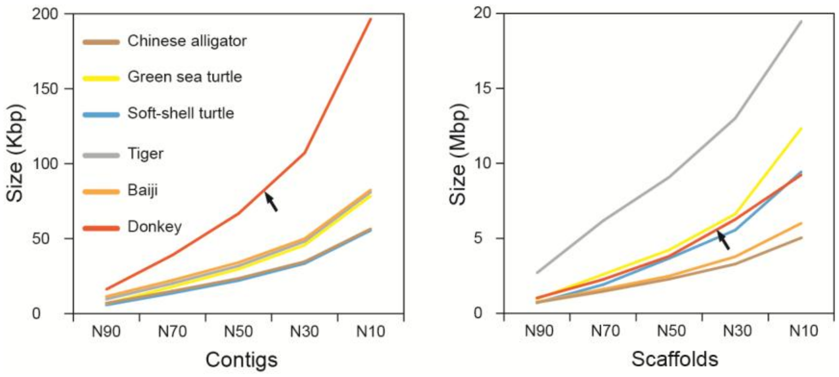

Supplementary Figure 2. Comparison of the sequence coverage and number of contigs and scaffolds for the donkey genome with other genomes. The arrow indicates the donkey genome.

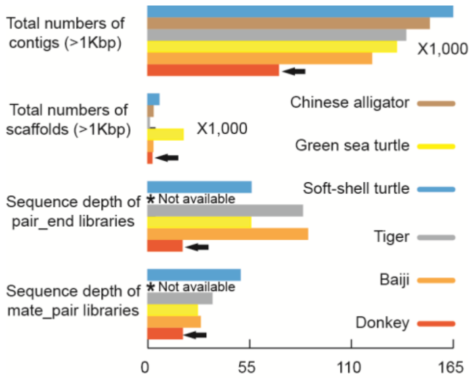

Supplementary Figure 3. Distribution of the transcriptome sequencing read lengths. RNA-seq was performed using the 454 FLX+ platform, and 1,390,416 reads were generated at an average length of 522 bp.

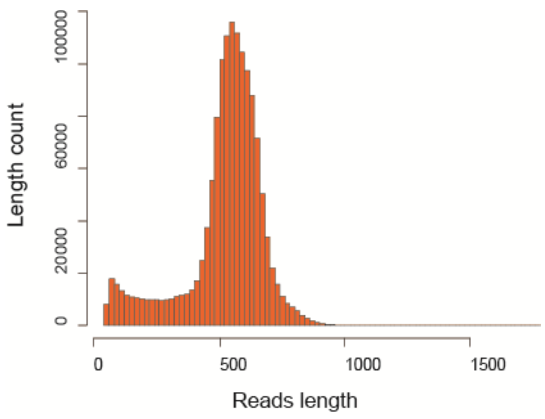

Supplementary Figure 4. KOG (EuKaryotic Orthologous Groups) assignments for donkey genes.

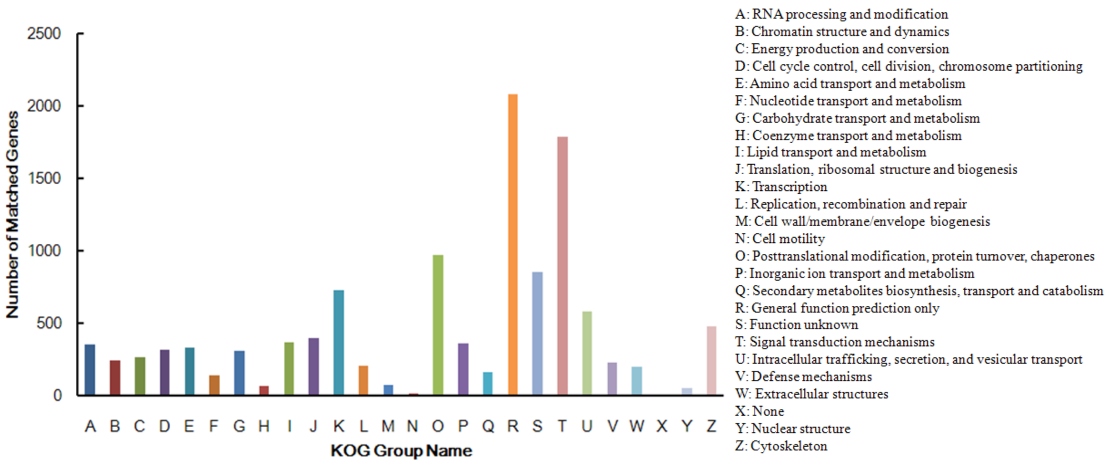

Supplementary Figure 5. KEGG (Kyoto Encyclopedia of Genes and Genomes) assignments for donkey genes.

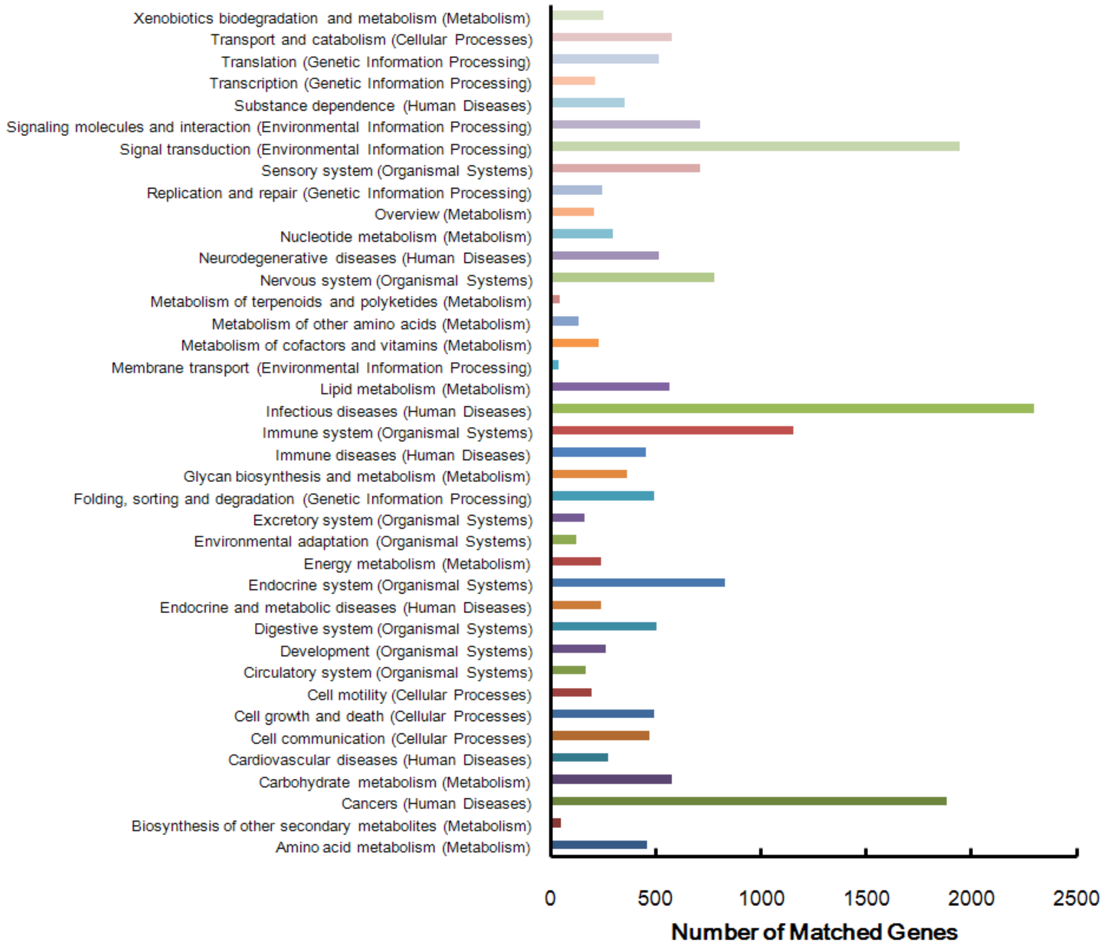

Supplementary Figure 6. RNA-seq Annotated genes in the donkey genome. (a), number of genes annotated according to the corresponding criteria; (b), number of genes confirmed by RNA-seq results.

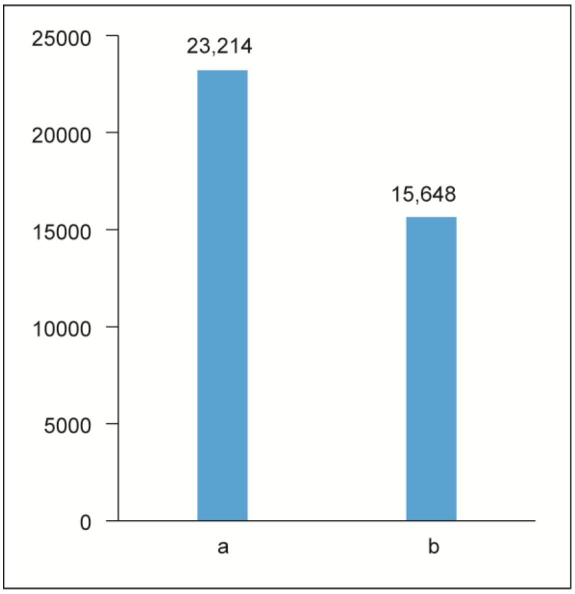

Supplementary Figure 7. Eight types of repetitive sequences in different regions (regions #1-6) of seven pairs of chromosomes.

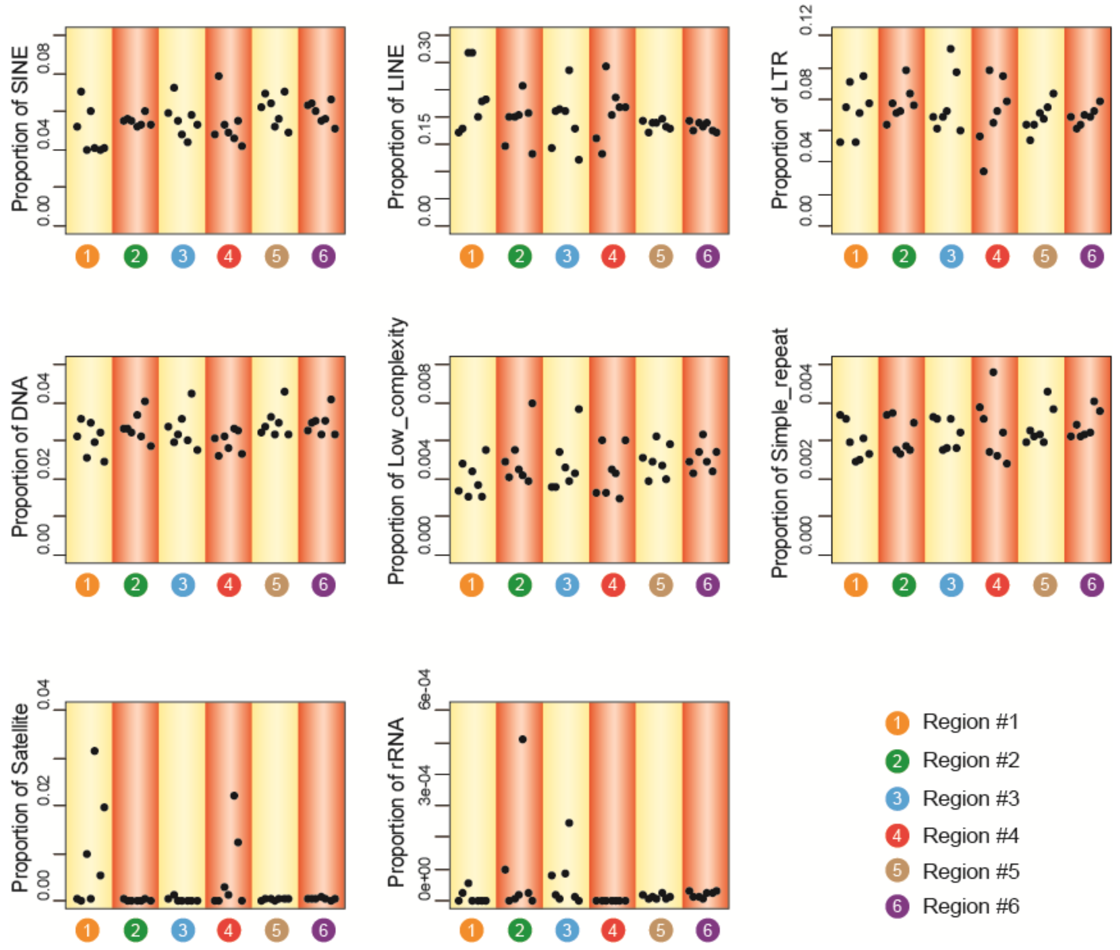

Supplementary Figure 8. Hierarchical clustering results of 20 types of satellite sequences.

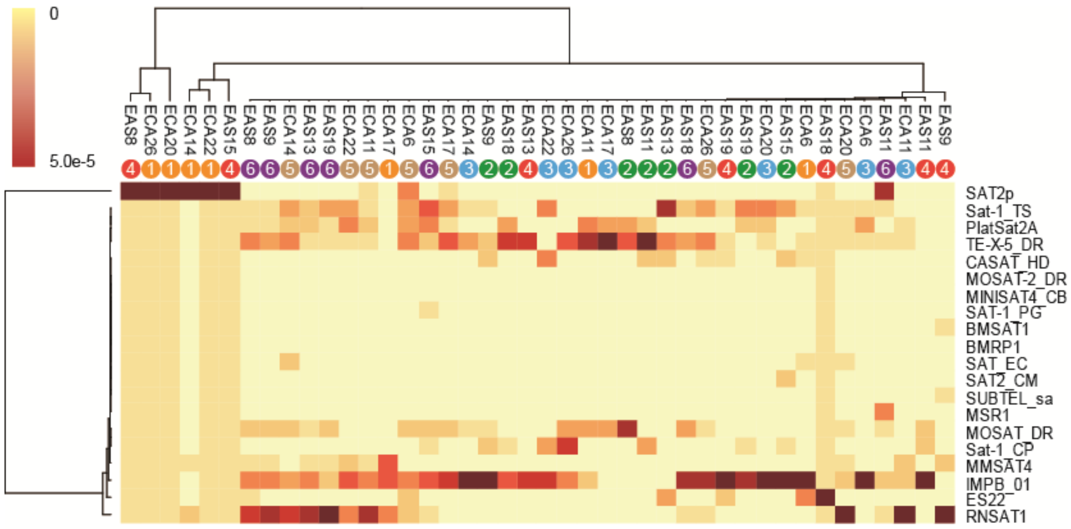

Supplementary Figure 9. Neighbor-joining tree for conservative 5SrRNA.

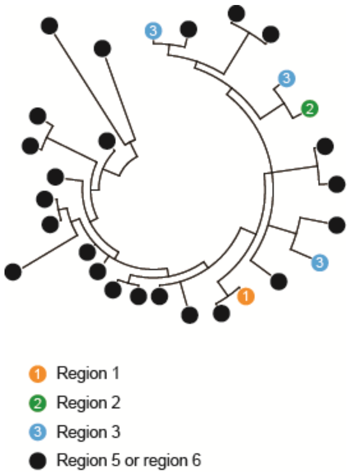

Supplementary Table 1. Genome sequencing summary for donkey. PE: Paired-end, standard genomic library sequenced using paired-end reads; SE: single-end; MP: Mate-paired.

Library Type	Number of library	Insert size	platform	Reads length	Raw data (bp)
PE400	1	400 bp	MiSeq	Paired-End, 2×251 bp	13,108,951,900
PE450	3	450 bp	MiSeq	Paired-End, 2×251 bp	31,738,312,460
PE700	3	700 bp	MiSeq	Paired-End, 2×251 bp	25,043,864,854
PE1000	1	1000 bp	MiSeq	Paired-End, 2×251 bp	3,971,185,456
SE1500	1	1500~1900 bp	454 FLX+	Single-End,average 510bp	389,938,235
MP3K	2	3000 bp	HiSeq2000	Mate-Paired, 2×100 bp	27,790,797,200
MP5K	2	5000 bp	HiSeq2000	Mate-Paired, 2×100 bp	24,393,026,800
MP8K	2	8000 bp	HiSeq2000	Mate-Paired, 2×100 bp	23,417,575,400
MP12K	1	12000 bp	HiSeq2000	Mate-Paired, 2×100 bp	10,020,707,400
MP15K	1	16000 bp	HiSeq2000	Mate-Paired, 2×100 bp	2,888,852,600

Supplementary Table 2. Qualified data for the donkey genome.

Library	clean data (bp)	Read1 length	Read2 length
PE400	10,321,705,614	242	219
PE450	21,732,933,747	231	196
PE700	16,246,446,690	230	184
PE1000	1,544,607,013	186	133
SE1500	389,938,235	Single-End, average 510bp	
MP3K	15,594,147,909	84	83
MP5K	14,258,628,782	86	85
MP8K	13,179,898,660	85	84
MP12K	5,877,899,778	85	84
MP15K	1,248,444,268	83	79

Supplementary Table 3. Genome sequencing summary for Asiatic wild ass.

Library Type	Number of library	Insert size	platform	Reads length	Raw data (bp)
PE500	1	500 bp	HiSeq2000	Paired-End, 2×100 bp	32,703,961,600

Supplementary Table 4. Qualified data for the Asiatic wild ass genome.

Library	clean data (bp)	Read1 length	Read2 length
PE500	28,556,773,659	97	96

Supplementary Table 5. Assembly results for the donkey genome.

	Contigs	Scaffolds
Min Sequence length (bp)	200	1,002
Max Sequence length (bp)	773,221	17,059,037
N10 (bp)	196,594	9,235,695
N30 (bp)	107,128	6,261,484
N50 (bp)	66,737	3,803,025
N70 (bp)	39,038	2,254,583
N90 (bp)	16,178	1,021,074
N number (bp)	0	33,114,414
Total Sequence length (bp)	2,324,805,719	2,357,920,133
Total Sequence Number	71,732	2,166

Supplementary Table 6. The completeness of 248 core eukaryotic genes in genome assemblies of donkey and other published genomes sequences using CEGMA.

Species	Complete(%)[a]	Partial(%)[b]
Donkey	57.26	92.34
Baiji	66.13	92.74
Chinese alligator	45.56	90.32
Tiger	52.42	90.32
Green sea turtle	37.90	86.69
Soft-shell turtle	34.68	83.87

[a] 'Complete' refers to predicted proteins in the set of 248 CEGs (Core Eukaryotic Genes) that could be aligned to the HMM of the KOG for that proteinfamily given alignment lengthsmore than 70% of the protein length.

[b]'Partial' refers to incomplete proteins that exceed a pre-computed minimum alignment score. A protein deemed to be 'Complete' will also be included in the 'Partial' set.

Supplementary Table 7. Summary of RNA-seq analysis of the female donkey.

Reads number	Average length	Raw data
1,390,416	522bp	725.8M

Supplementary Table 8. Number of heterozygous (within an individual) SNPs and Indels in the donkey and Asiatic wild ass genomes.

	donkey	Asiatic wild ass
Heterozygous SNP	2,187,070	3,321,087
Heterozygous INDEL	247,822	213,735

Supplementary Table 9. Enrichment analysis of expansive gene families in the donkey genome. (MF: molecular function; CC: cell component; BP: biological process)

Functional category	Term ID	Term description	Involved gene number	P_values
BP	GO:0050911	detection of chemical stimulus involved in sensory perception of smell	34	3.2E-11
BP	GO:0007186	G-protein coupled receptor signaling pathway	38	1.54E-09
BP	GO:0044699	single-organism process	4	0.000956
BP	GO:0007608	sensory perception of smell	3	0.006588
BP	GO:0035023	regulation of Rho protein signal transduction	4	0.01058
BP	GO:0022617	extracellular matrix disassembly	3	0.01104
BP	GO:0030509	BMP signaling pathway	3	0.01853
BP	GO:0007411	axon guidance	6	0.01889
BP	GO:0006805	xenobiotic metabolic process	3	0.02215
BP	GO:0001501	skeletal system development	3	0.0428
BP	GO:0008152	metabolic process	5	0.0466
CC	GO:0005634	nucleus	18	0.000362
CC	GO:0005604	basement membrane	5	0.00221
CC	GO:0005796	Golgi lumen	4	0.002335
CC	GO:0005886	plasma membrane	47	0.002908
CC	GO:0070062	extracellular vesicular exosome	5	0.003173
CC	GO:0016021	integral to membrane	63	0.01051
CC	GO:0043202	lysosomal lumen	3	0.01686
CC	GO:0030018	Z disc	4	0.01866
CC	GO:0019898	extrinsic to membrane	3	0.02825
MF	GO:0004984	olfactory receptor activity	36	3.52E-12
MF	GO:0004930	G-protein coupled receptor activity	39	1.39E-11
MF	GO:0005200	structural constituent of cytoskeleton	4	0.004218
MF	GO:0005488	binding	4	0.005734
MF	GO:0005201	extracellular matrix structural constituent	3	0.006588
MF	GO:0016887	ATPase activity	4	0.01143
MF	GO:0005089	Rho guanyl-nucleotide exchange factor activity	4	0.01636
MF	GO:0005515	protein binding	11	0.02146
MF	GO:0005509	calcium ion binding	15	0.02923
MF	GO:0005516	calmodulin binding	4	0.04014
MF	GO:0004725	protein tyrosine phosphatase activity	4	0.04205
KEGG	map04740	Olfactory transduction	26	5.36E-08
KEGG	map04974	Protein digestion and absorption	4	0.01327
KEGG	map04512	ECM-receptor interaction	3	0.02613
KEGG	map05320	Autoimmune thyroid disease	3	0.02825
KEGG	map05152	Tuberculosis	6	0.0288
KEGG	map05146	Amoebiasis	4	0.03301

Supplementary Table 10. Enrichment analysis of expansive gene families in the Thoroughbred horse genome. (MF: molecular function; BP: biological process)

Functional category	Term ID	Term description	Involved gene number	P_values
BP	GO:0006952	defense response	3	0.011694
BP	GO:0006950	response to stress	3	0.028023
BP	GO:0006953	acute-phase response	2	0.03524
BP	GO:0002526	acute inflammatory response	2	0.041051
MF	GO:0004857	enzyme inhibitor activity	2	0.025568
MF	GO:0030234	enzyme regulator activity	2	0.045455
KEGG	ecb04740	Olfactory transduction	17	4.28E-13
KEGG	ecb05322	Systemic lupus erythematosus	6	7.15E-05

Supplementary Table 11. Enrichment analysis of rapidly evolving genes in the donkey genome. (BP:biological process)

Functional category	Term ID	Term description	Mean of ω (Donkey)	Mean of ω (Horse)	p_values	Gene number	Fold change in mean ω (Donkey) vs. ω (Horse)
BP	GO:0009060	aerobic respiration	0.468311429	0.212797	0.027965	7	2.200741149
BP	GO:0030900	forebrain development	0.4581714	0.216707	0.00671	20	2.114239754
BP	GO:0045619	regulation of lymphocyte differentiation	0.500157083	0.250011	0.02467	10	2.000538309
BP	GO:0006099	tricarboxylic acid cycle	0.459216364	0.246425	0.016761	6	1.863510263
BP	GO:0046356	acetyl-CoA catabolic process	0.459216364	0.246425	0.016761	6	1.863510263
BP	GO:0006357	regulation of transcription from RNA polymerase II promoter	0.309431321	0.166096	0.016169	65	1.862962067
BP	GO:0007050	cell cycle arrest	0.34912775	0.188365	0.020903	14	1.853461559
BP	GO:0032774	RNA biosynthetic process	0.297064828	0.162725	0.043553	29	1.825568057
BP	GO:0045944	positive regulation of transcription from RNA polymerase II promoter	0.254304259	0.144119	0.027416	36	1.764542694
BP	GO:0006417	regulation of translation	0.393015333	0.2268	0.010715	18	1.732873538
BP	GO:0051254	positive regulation of RNA metabolic process	0.261971181	0.154286	0.010306	47	1.69796081
BP	GO:0045893	positive regulation of transcription, DNA-dependent	0.262990559	0.155364	0.013977	46	1.692738235
BP	GO:0010608	posttranscriptional regulation of gene expression	0.359831739	0.224674	0.015445	24	1.601574328
BP	GO:0051187	cofactor catabolic process	0.359052	0.225092	0.015928	7	1.595134434
BP	GO:0000723	telomere maintenance	0.469709375	0.300447	0.002098	8	1.563369148
BP	GO:0070727	cellular macromolecule localization	0.373786111	0.239228	0.048083	38	1.562466928
BP	GO:0017148	negative regulation of translation	0.462737857	0.300145	0.006696	8	1.541714362
BP	GO:0048534	hemopoietic or lymphoid organ development	0.363756941	0.239752	0.002764	31	1.517220228
BP	GO:0030097	hemopoiesis	0.394863067	0.26126	0.001245	30	1.51137741
BP	GO:0007548	sex differentiation	0.424734118	0.287558	0.002834	21	1.477039898
BP	GO:0002520	immune system development	0.346672447	0.236993	0.003585	32	1.462798246

BP	GO:0006281	DNA repair	0.396609732	0.276214	0.005421	32	1.435877436
BP	GO:0048608	reproductive structure development	0.430420952	0.30198	0.004686	18	1.425327087
BP	GO:0045137	development of primary sexual characteristics	0.429110476	0.305384	0.005081	18	1.405149172
BP	GO:0008406	gonad development	0.453081316	0.329392	0.008153	16	1.375508613
BP	GO:0015031	protein transport	0.294934756	0.214471	0.038238	65	1.375176152
BP	GO:0045184	establishment of protein localization	0.294226721	0.214088	0.044942	65	1.374324411
BP	GO:0032268	regulation of cellular protein metabolic process	0.318470519	0.231948	0.041033	43	1.373023745
BP	GO:0032504	multicellular organism reproduction	0.344703741	0.253658	0.019033	46	1.358928849
BP	GO:0048609	reproductive process in a multicellular organism	0.344703741	0.253658	0.019033	46	1.358928849
BP	GO:0006974	response to DNA damage stimulus	0.35513028	0.261345	0.029436	36	1.358856405
BP	GO:0009968	negative regulation of signal transduction	0.251353462	0.188143	0.02522	24	1.335968851
BP	GO:0065003	macromolecular complex assembly	0.298143231	0.225823	0.035578	58	1.320253487
BP	GO:0008585	female gonad development	0.470596667	0.358097	0.010106	11	1.314160981
BP	GO:0043933	macromolecular complex subunit organization	0.294688915	0.225482	0.039921	61	1.306926975
BP	GO:0006259	DNA metabolic process	0.344672128	0.264876	0.023344	47	1.301256548
BP	GO:0033554	cellular response to stress	0.331512551	0.257648	0.042435	50	1.286689072
BP	GO:0046545	development of primary female sexual characteristics	0.4517768	0.352777	0.016781	11	1.280630699
BP	GO:0046660	female sex differentiation	0.4517768	0.352777	0.016781	11	1.280630699
BP	GO:0022613	ribonucleoprotein complex biogenesis	0.267161806	0.209294	0.004872	23	1.276492038
BP	GO:0003006	reproductive developmental process	0.352671519	0.279023	0.030695	27	1.263949535
BP	GO:0032200	telomere organization	0.451973529	0.360221	0.002607	8	1.254713207
BP	GO:0006284	base-excision repair	0.346288824	0.277465	0.032216	6	1.24804374
BP	GO:0006730	one-carbon metabolic process	0.389817222	0.317026	0.018309	15	1.229608198
BP	GO:0006396	RNA processing	0.285309672	0.233234	0.017031	51	1.223277994
BP	GO:0006446	regulation of translational initiation	0.470618	0.398308	0.0186	8	1.181542927
BP	GO:0021537	telencephalon development	0.33231	0.281946	0.029594	10	1.17862995

BP	GO:0022402	cell cycle process	0.34776256	0.31212	0.002263	57	1.114196606
BP	GO:0007049	cell cycle	0.334022819	0.301825	0.010578	70	1.106676614
BP	GO:0051090	regulation of transcription factor activity	0.302910278	0.273746	0.044262	13	1.106536159
BP	GO:0022602	ovulation cycle process	0.369524091	0.33527	0.008096	11	1.102170167
BP	GO:0021987	cerebral cortex development	0.423459412	0.391195	0.005638	8	1.082477358
BP	GO:0021543	pallium development	0.339452273	0.31452	0.033161	8	1.079269306
BP	GO:0000087	M phase of mitotic cell cycle	0.373287273	0.347318	0.016474	25	1.074771021
BP	GO:0000280	nuclear division	0.382009176	0.359573	0.02407	24	1.062397591
BP	GO:0007067	mitosis	0.382009176	0.359573	0.02407	24	1.062397591
BP	GO:0048285	organelle fission	0.3691325	0.353044	0.036003	24	1.045571211
BP	GO:0000278	mitotic cell cycle	0.334097154	0.321872	0.026409	36	1.037982159
BP	GO:0000279	M phase	0.38629	0.3752	0.014312	34	1.029558667
MF	GO:0003702	RNA polymerase II transcription factor activity	0.425302785	0.129383	0.02382	26	3.287173155
MF	GO:0003714	transcription corepressor activity	0.381889412	0.143207	0.034927	17	2.666694051
MF	GO:0003712	transcription cofactor activity	0.327390175	0.170799	0.007749	38	1.916816119
MF	GO:0008134	transcription factor binding	0.306913459	0.173941	0.019749	48	1.764473792
MF	GO:0043565	sequence-specific DNA binding	0.317935251	0.186359	0.043653	53	1.706033969
MF	GO:0005504	fatty acid binding	0.355241333	0.218435	0.012361	8	1.626304738
MF	GO:0046983	protein dimerization activity	0.288388679	0.196181	0.04488	48	1.470012311
MF	GO:0016563	transcription activator activity	0.265449921	0.183474	0.042536	38	1.446800697
MF	GO:0008373	sialyltransferase activity	0.506252	0.396232	0.041709	5	1.27766561
MF	GO:0004527	exonuclease activity	0.330923214	0.268063	0.024515	9	1.234500217
MF	GO:0004518	nuclease activity	0.351341695	0.294962	0.037417	18	1.191142095
MF	GO:0019904	protein domain specific binding	0.268009897	0.231765	0.037055	20	1.156388727

Supplementary Table 12. Enrichment analysis of rapidly evolving genes in the Thoroughbred horse genome. (MF: molecular function; BP: biological process)

Functional category	Term ID	Term description	Mean of ω (Donkey)	Mean of ω (Horse)	p_values	Gene number	Fold change in mean ω (Horse) vs. ω (Donkey)
BP	GO:0042254	ribosome biogenesis	0.187661667	0.353428	0.035248	15	1.883327264
BP	GO:0045494	photoreceptor cell maintenance	0.282578333	0.510645	0.041551	4	1.807091839
BP	GO:0021532	neural tube patterning	0.128933	0.219653	0.041551	4	1.703621261
BP	GO:0001947	heart looping	0.271165833	0.407191	0.046821	4	1.501630306
BP	GO:0019932	second-messenger-mediated signaling	0.183038831	0.246639	0.001296	20	1.347467254
BP	GO:0008654	phospholipid biosynthetic process	0.226798857	0.284659	0.036526	9	1.255115924
BP	GO:0051345	positive regulation of hydrolase activity	0.236316842	0.296162	0.00288	16	1.253243118
BP	GO:0051348	negative regulation of transferase activity	0.192748286	0.240657	0.026713	9	1.24855511
BP	GO:0051640	organelle localization	0.253836875	0.316034	0.021295	9	1.245026949
BP	GO:0060041	retina development in camera-type eye	0.30531375	0.357851	0.040076	5	1.172077085
BP	GO:0006916	anti-apoptosis	0.364841875	0.421109	0.044915	14	1.154223491
BP	GO:0006289	nucleotide-excision repair	0.30892619	0.356283	0.016082	7	1.153295979
BP	GO:0043086	negative regulation of catalytic activity	0.214188333	0.247005	0.007771	20	1.153214072
BP	GO:0007030	Golgi organization	0.287156364	0.330975	0.041551	4	1.152593456
BP	GO:0009062	fatty acid catabolic process	0.256997333	0.289177	0.043807	5	1.125215307
BP	GO:0010498	proteasomal protein catabolic process	0.288727838	0.317876	0.036526	9	1.100955449
BP	GO:0043161	proteasomal ubiquitin-dependent protein catabolic process	0.288727838	0.317876	0.036526	9	1.100955449
BP	GO:0016042	lipid catabolic process	0.252559385	0.276944	0.005337	15	1.096550027
BP	GO:0051336	regulation of hydrolase activity	0.238613131	0.251643	0.027565	21	1.054608491
BP	GO:0051321	meiotic cell cycle	0.413962143	0.431993	0.004068	11	1.043557008
BP	GO:0048536	spleen development	0.25211	0.26125	0.031969	4	1.036254016
BP	GO:0006913	nucleocytoplasmic transport	0.2808754	0.290537	0.031725	12	1.034399595
BP	GO:0051169	nuclear transport	0.276276275	0.285749	0.034355	12	1.034286509
BP	GO:0022403	cell cycle phase	0.362079272	0.364713	3.37E-05	34	1.007274028
MF	GO:0008227	amine receptor activity	0.115142632	0.183432	0.016507	6	1.593085921

MF	GO:0005543	phospholipid binding	0.230389107	0.333719	0.030285	13	1.448503657

Supplementary Table 13. Repeat sequences in the donkey genome.

	Number of elements	Length occupied	Percentage of sequence
SINEs:	964330	175869828bp	7.46%
Alu/B1	0	0bp	0.00%
MIRs	560212	86172367bp	3.65%
LINEs:	934805	539350954bp	22.87%
LINE1	516000	413293333bp	17.53%
LINE2	357376	109854592bp	4.66%
L3/CR1	48393	11794134bp	0.50%
RTE	12552	4285769bp	0.18%
LTR elements:	380349	170193797bp	7.22%
ERVL	107069	53651613bp	2.28%
ERVL-MaLRs	175016	65729822bp	2.79%
ERV_classI	72276	42697897bp	1.81%
ERV_classII	1747	1173838bp	0.05%
DNA elements:	388813	81092370bp	3.44%
hAT-Charlie	224818	45590121bp	1.93%
TcMar-Tigger	75515	17447332bp	0.74%
Satellites:	422	1247289bp	0.05%
Simple repeats:	198517	10559725bp	0.45%
Low complexity:	265131	11227865bp	0.48%

Supplementary Table 14. Pericentromeric regions in the Thoroughbred horse and donkey genomes.

Chromosomes of Thoroughbred horse	Region #1 (Thoroughbred horse)	Region #2 (donkey)	Region #3 (Thoroughbred horse)	Region #4 (donkey)
6	20M-30M	scaffold1449:0-21669;<br>scaffold213:0-1335911;<br>scaffold328:0-2333015;<br>scaffold388:1072241-2001066	5.5M-9M	scaffold200:0-3629144;<br>scaffold355:0-2187355;<br>scaffold50:4049253-7518837;
11	26M-30M	scaffold491:0-1546321;<br>scaffold66:4865946-6958850	45M-51M	scaffold486:0-210156;<br>scaffold9:6066945-9526757;<br>scaffold976:0-312072
14	0-4M	scaffold1807:0-1449;<br>scaffold219:0-1703000;<br>scaffold394:1319215-1971954;<br>scaffold45:0-7860578	43M-53M	scaffold1342:0-51966;<br>scaffold1462:0-19272;<br>scaffold1633:0-3551;<br>scaffold481:0-1589768;<br>scaffold848:0-508249;<br>scaffold864:0-481037;<br>scaffold918:0-402223
17	0-4M	scaffold110:1468912-5198093;<br>scaffold783:0-45959;<br>scaffold830:396350-542364	10M-14M	scaffold1587:0-5766;<br>scaffold206:0-811194
20	0-4M	scaffold1017:0-261445;<br>scaffold1033:0-240055;<br>scaffold1112:0-170579;<br>scaffold1188:0-119121;<br>scaffold1254:0-86521;<br>scaffold1264:0-81445;<br>scaffold1345:0-49439;<br>scaffold1534:0-9464;<br>scaffold1543:0-8915;<br>scaffold18:4145634-4801528;<br>scaffold244:0-1002888;<br>scaffold818:0-568079;<br>scaffold951:0-342753;<br>scaffold986:0-303444	30M-34M	scaffold450:0-1720145;<br>scaffold615:0-1105635;<br>scaffold750:0-719256;<br>scaffold838:0-533065
22	0-4M	scaffold606:0-1138699;<br>scaffold71:0-4364170;<br>scaffold71:6704483-6776457;<br>scaffold867:0-474124	13.5M-19.5M	scaffold1000:0-284273;<br>scaffold117:810785-1183002;<br>scaffold1395:0-34955;<br>scaffold20:8467469-9235695;<br>scaffold28:852521-858424;<br>scaffold413:0-1876501;<br>scaffold712:0-811768;

				scaffold905:0-419880
26	0-4M	scaffold62:2583134-6478310	10M-14M	scaffold1133:0-152466;<br>scaffold1229:0-97236;<br>scaffold123:1539637-2102274;<br>scaffold344:1045554-2246623;<br>scaffold714:0-804170;<br>scaffold826:0-551156

Supplementary Table 15. Noncoding sequences in the donkey genome.

ncRNA Type	Copy	Average length (bp)	Total length (bp)
snoRNA	512	110	56165
CD-box	284	86	24408
HACA-box	207	135	28044
scaRNA	21	177	3713
miRNA	1198	89	84673
snRNA	530	111	58745
Cis-reg	172	79	13578
lncRNA	189	147	27819
Other	256	239	61209
Summary	3590	109	390402

Supplementary Table 16. Enrichment analysis of target genes from expanded miRNA families in the donkey genome.

Term description	Involved gene number	P_values
Neurotrophin signaling pathway	29	9.11E-07
Ubiquitin mediated proteolysis	29	7.33E-06
Chronic myeloid leukemia	20	9.25E-06
ErbB signaling pathway	21	2.57E-05
Endometrial cancer	15	6.83E-05
Pathways in cancer	49	8.44E-05
Insulin signaling pathway	26	1.27E-04
Glioma	16	1.74E-04
Prostate cancer	19	3.67E-04
Colorectal cancer	18	5.27E-04
Renal cell carcinoma	16	5.91E-04
Fc gamma R-mediated phagocytosis	19	8.36E-04
Acute myeloid leukemia	14	8.68E-04
Focal adhesion	31	0.001361967
Type II diabetes mellitus	12	0.001474374
MAPK signaling pathway	38	0.001590571
Aldosterone-regulated sodium reabsorption	11	0.001722184
Wnt signaling pathway	24	0.003721774
Non-small cell lung cancer	12	0.00472718
Progesterone-mediated oocyte maturation	16	0.005083663
Axon guidance	21	0.005416773
Pancreatic cancer	14	0.006565047
Melanogenesis	17	0.008251476
mTOR signaling pathway	11	0.010503824
Long-term potentiation	13	0.010621187
Adherens junction	14	0.011557011
Melanoma	13	0.014832715
Endocytosis	25	0.020519368
VEGF signaling pathway	13	0.022284553
Oocyte meiosis	16	0.042579033
Hedgehog signaling pathway	10	0.043454872
Small cell lung cancer	13	0.048597727

Supplementary Table 17. Summary of small RNA-seq analysis of the female donkey.

Annotation	Unique sequences
rRNA	17514
tRNA	4635
snoRNA	3943
miRNA	22100
Intron	1130

Supplementary Table 18. Novel miRNA identified in the donkey genome.

miRNA	Sequences	Abundance
eas-m0001-5p	CTGTAAACACCCGACTGGAAG	2113
eas-m0002-5p	GCTTGGCACCTCGTAAGCACTC	1916
eas-m0003-5p	AGAGATCAGAGGCGCAGAGTG	593
eas-m0004-3p	ACCTTGCGCTACTCAGGTCTGCT	579
eas-m0005-3p	TCACTCCTCTCCTCCCGTCTTC	384
eas-m0006-5p	CTGCCCCAAGAAGCCTGGACTTG	250
eas-m0007-3p	ATTCAACGGGTCTTTATTGAG	230
eas-m0008-3p	CGGGGCCGCCCCCGCGGGCT	198
eas-m0009-3p	AGTCGGTGCCATCTGGGTAGAG	161
eas-m0010-3p	AGTCGGTGCCATCTGGGTAGAG	161
eas-m0011-3p	CCAGTTACCGCTTCCGCTACCG	129
eas-m0012-3p	GGGGCTGACCCAATGGCACAGT	108
eas-m0013-3p	ACGGGCTGCAGCGCCGACGCC	93
eas-m0014-5p	GGCTCTGTGAGGTCGGCTCAA	92
eas-m0015-3p	TGCCTGGGCCTCTGGAACCA	72
eas-m0016-5p	CTGTCAACCATCCAGCTGTTTG	71
eas-m0017-3p	TAAATGGTCCCCTAGTGCTCTG	50
eas-m0018-5p	GTTGATCAGAGAACATACATTT	47
eas-m0019-3p	ATTCGGCACCTTCTGGGTAGCG	39
eas-m0020-5p	TGTACATGGTAGGCTTTCATTC	31
eas-m0021-5p	TTTCCCCTAGTTCTCTTGCATA	28
eas-m0022-5p	TTCCTCTCTGCCCCATAGGGTGT	26
eas-m0023-3p	CTGCTGAGCTAGCACTTCCCGAG	25
eas-m0024-3p	GTTGCAGCTGCCTGGGAGTGATT	22
eas-m0025-3p	CTTGCCCTTCTTCCTCTGAC	21
eas-m0026-5p	ACTTTTTGGTCTGAGTGCGTCC	17
eas-m0027-3p	TTTTCGCCGTCTGAGTCCGCCG	15
eas-m0028-5p	ATTTCTGGACAAATGCTCCCTGC	14
eas-m0029-5p	CAAGGATTCAACCAACTTCGGA	14
eas-m0030-3p	TCAACAGACATTAATTGGGCGCC	14
eas-m0031-3p	GCCAGCTTGAGCCCACCTACATA	13
eas-m0032-3p	ACCCATATACCCCTCGGATACA	13
eas-m0033-3p	TTCCGTCCCCCACTCGCCTCCTG	12
eas-m0034-5p	CTAGTAGGTGTTCAGTAAGTGT	12
eas-m0035-5p	GTCTCTGGTTCTTGTCGTAC	12
eas-m0036-5p	TATGGAACTTCAGACAATAGGGC	12
eas-m0037-5p	CTGTTGGCCTTTGGACTGGAA	11
eas-m0038-5p	GGCAGGGCTGAGACCTGGACTG	11
eas-m0039-3p	CTGCACTTGAAGCTGAGCCTG	11
eas-m0040-3p	CTGCCCACCTGTCACCTGCCCTG	10

Supplementary Table 19. Enrichment analysis of target genes of novel miRNAs identified in the donkey genome.

eas-m0001-5p		
**Term description**	**Involved gene number**	**P_values**
Cell cycle	19	0.003777517
Axon guidance	19	0.003777517
Progesterone-mediated oocyte maturation	15	0.003954445
Phosphatidylinositol signaling system	13	0.007166977
Oocyte meiosis	16	0.010888807
Inositol phosphate metabolism	10	0.019918559
Fc epsilon RI signaling pathway	12	0.022480045
Pathways in cancer	33	0.023978817
Dilated cardiomyopathy	12	0.029576939
Regulation of actin cytoskeleton	23	0.030818307
Toll-like receptor signaling pathway	13	0.036258183
Pancreatic cancer	11	0.0364988
Small cell lung cancer	12	0.038152009
Hypertrophic cardiomyopathy (HCM)	11	0.047178673
Fc gamma R-mediated phagocytosis	12	0.048329925
Arrhythmogenic right ventricular cardiomyopathy (ARVC)	10	0.063468053
Colorectal cancer	11	0.064435642
MAPK signaling pathway	27	0.065649692
Insulin signaling pathway	16	0.075517024
Endometrial cancer	8	0.076494069
Focal adhesion	21	0.079113204
Wnt signaling pathway	15	0.082256427
Tight junction	15	0.0867579
Non-small cell lung cancer	8	0.091031401
Ubiquitin mediated proteolysis	15	0.091412913
Neurotrophin signaling pathway	15	0.096221864
**eas-m0016-5p**		
**Term description**	**Involved gene number**	**P_values**
Long-term potentiation	13	0.001378065
Renal cell carcinoma	12	0.003009046
ErbB signaling pathway	14	0.003287375
GnRH signaling pathway	14	0.00679383
Progesterone-mediated oocyte maturation	13	0.009853494
Insulin signaling pathway	17	0.015168116
Vascular smooth muscle contraction	14	0.017674888
Ubiquitin mediated proteolysis	16	0.01980017
Gap junction	12	0.02228724
Adipocytokine signaling pathway	10	0.025114651

Starch and sucrose metabolism	7	0.026855112
MAPK signaling pathway	26	0.03094091
Long-term depression	10	0.033050373
Calcium signaling pathway	18	0.033397315
Oocyte meiosis	13	0.044055391
Prostate cancer	11	0.047093949
mTOR signaling pathway	8	0.049203698
Phosphatidylinositol signaling system	10	0.049860494
Glioma	9	0.052277234
Apoptosis	11	0.054250943
Cell cycle	14	0.058345612
Pathways in cancer	28	0.060085423
Regulation of autophagy	5	0.069761567
Acute myeloid leukemia	8	0.070011686
Dilated cardiomyopathy	10	0.071523731
**eas-m0026-5p**		
**Term description**	**Involved gene number**	**P_values**
Arrhythmogenic right ventricular cardiomyopathy (ARVC)	22	0.001035209
Lysine degradation	15	0.001293655
Viral myocarditis	19	0.001974023
ECM-receptor interaction	22	0.011386531
Purine metabolism	34	0.013871071
Dilated cardiomyopathy	21	0.014841824
Hypertrophic cardiomyopathy (HCM)	20	0.01668494
Cell cycle	29	0.020138058
TGF-beta signaling pathway	20	0.02554295
Adherens junction	18	0.037288349
Tight junction	28	0.050349253
Oocyte meiosis	24	0.055208192
ErbB signaling pathway	20	0.059477187
Spliceosome	27	0.067245732
Focal adhesion	39	0.067366587
Calcium signaling pathway	33	0.076447739
Regulation of actin cytoskeleton	39	0.076963619
Alanine,aspartate and glutamate metabolism	9	0.087357931
Axon guidance	26	0.088707488
Phosphatidylinositol signaling system	17	0.095772967
**eas-m0028-5p**		
**Term description**	**Involved gene number**	**P_values**
Pathways in cancer	81	0.00203275
Hypertrophic cardiomyopathy (HCM)	25	0.006917152
Dilated cardiomyopathy	26	0.007219962
Viral myocarditis	19	0.025235539

Oocyte meiosis	30	0.034415761
Arrhythmogenic right ventricular cardiomyopathy (ARVC)	21	0.036532845
Aminoacyl-tRNA biosynthesis	14	0.045881186
Insulin signaling pathway	36	0.047876863
Pancreatic cancer	21	0.056727446
Cell adhesion molecules (CAMs)	33	0.057029293
Lysine degradation	13	0.065338637
Tryptophan metabolism	14	0.066480641
Mismatch repair	9	0.067457113
Butanoate metabolism	11	0.091084451
Jak-STAT signaling pathway	33	0.092000511
**eas-m0035-5p**		
**Term description**	**Involved gene number**	**P_values**
Calcium signaling pathway	48	0.001628761
ErbB signaling pathway	26	0.017741458
ABC transporters	15	0.018817179
Axon guidance	34	0.02797493
Purine metabolism	39	0.031169087
ECM-receptor interaction	24	0.037540082
Focal adhesion	49	0.037973594
Long-term potentiation	20	0.059922542
Insulin signaling pathway	35	0.063812474
Oocyte meiosis	28	0.078933815
Endocytosis	46	0.080488547
Dorso-ventral axis formation	9	0.080982595
Fatty acid metabolism	12	0.086577966
Aminoacyl-tRNA biosynthesis	13	0.086640066
Regulation of actin cytoskeleton	47	0.090198103
Valine,leucine and isoleucine biosynthesis	5	0.092771473
